# LncRNA HEPFAL accelerates ferroptosis in hepatocellular carcinoma by regulating SLC7A11 ubiquitination

**DOI:** 10.1038/s41419-022-05173-1

**Published:** 2022-08-25

**Authors:** Baofu Zhang, Wenming Bao, Sina Zhang, Bo Chen, Xiang Zhou, Jungang Zhao, Zhehao Shi, Tan Zhang, Ziyan Chen, Luhui Wang, Xiangtao Zheng, Gang Chen, Yi Wang

**Affiliations:** 1 Department of Hepatobiliary Surgery, The First Affiliated Hospital of Wenzhou Medical University, Wenzhou, China; 2 Key Laboratory of Diagnosis and Treatment of Severe Hepato-Pancreatic Diseases of Zhejiang Province, The First Affiliated Hospital of Wenzhou Medical University, Wenzhou, Zhejiang China; 3 Department of Vascular Surgery, The Second Affiliated Hospital of Wenzhou Medical University, Wenzhou, China; 4 Department of Epidemiology and Biostatistics, School of Public Health and Management, Wenzhou Medical University, Wenzhou, Zhejiang China

**Keywords:** Liver cancer, Cell death

## Abstract

Ferroptosis is a new type of cell death that has been recognized in recent years and is different from apoptosis, autophagy, and necrosis. It is mainly due to cellular iron homeostasis and lipid peroxidation of iron metabolism caused by large accumulation. There is a close correlation between ferroptosis and hepatocellular carcinoma (HCC). This study shows that the expression of the long noncoding RNA HEPFAL was reduced in HCC tissues. We found that lncRNA HEPFAL can promote ferroptosis by reducing the expression of solute carrier family 7 member 11 (SLC7A11) and increasing the levels of lipid reactive oxygen species (ROS) and iron (two surrogate markers of ferroptosis). In addition, we found that lncRNA HEPFAL increases the sensitivity of erastin-induced ferroptosis, which may be related to mTORC1, and lncRNA HEPFAL can promote the ubiquitination of SLC7A11 and reduce the stability of the SLC7A11 protein, resulting in decreased expression. Understanding these mechanisms indicates that lncRNAs related to ferroptosis are essential for the occurrence and treatment of HCC.

## Introduction

The most common type of primary liver cancer in the world is hepatocellular carcinoma (HCC), which accounts for more than 80% of all cases. Liver cancer is the sixth most common cancer in the world and the fourth most common cancer cause of death [1]. Although significant progress has been made in the diagnosis and treatment of liver cancer and the global age-standardized incidence rate of liver cancer is slowly decreasing, the overall burden of liver cancer worldwide has increased over time, and the five-year survival rate is low at 12% [2]. The development mechanisms of HCC have gradually been discovered, and researchers have made great progress in characterizing cancer heterogeneity [3].

Long noncoding RNA is a type of transcript encoded by the genome, but most lncRNAs do not translate into protein [4]. LncRNAs in cancer mutations and disorders play a vital role in the occurrence and development of cancer. For example, lncRNAs PCA3, PCGEM1, and PCAT-1 are highly specific to prostate cancer, while more lncRNAs involved in lung cancer [5]. Studies have also shown that in HCC, lncRNAs play an important role. Various proteins related to HCC exhibit abnormal expression and interact with lncRNAs to regulate the cancer phenotype [6]. Therefore, lncRNAs have great potential as new cancer markers and therapeutic targets.

Ferroptosis is a new type of iron-dependent programmed cell death discovered by Dixon et al. [7], which is different from other forms of cell death and mainly manifests with high mitochondrial density. The cystine-glutamate antiporter system (system Xc-) and glutathione peroxidase 4 (GPX4) are critical regulators of ferroptosis [8]. System Xc- can maintain the activity of GPX4 through cystine transport [9, 10]. When ferroptosis occurs, glutathione (GSH) in the cell is consumed, leading to the inactivation of GPX4, which ultimately leads to lipid peroxidation and the production of a large number of reactive oxygen species, constituting the main mechanism leading to cell ferroptosis [11–13]. As an essential part of the Xc- system, SLC7A11 has been confirmed to be highly expressed in some cancer cells and can attenuate the ferroptosis effect caused by erastin [14]. In HCC, erastin has been verified to have anti-tumor effects, and SLC7A11 is closely related to the occurrence and development of HCC [15].

Ferroptosis plays a vital role in the occurrence and development of cancer, but its molecular mechanism in epigenetics is not yet fully understood. Ferroptosis has been confirmed to be associated with oncogenes, such as RAS and TP53 [8, 16]. Studies have shown that LINC00336 can inhibit ferroptosis in lung cancer by acting as a competitive endogenous RNA [17], LINC00618 can reduce the expression of SLC7A11 by interacting with LSH, which leads to ferroptosis of cells [18], and lncRNA P53RRA in the cytoplasm can promote the occurrence of ferroptosis by activating the P53 pathway [19]. Currently, studies on the regulation of ferroptosis by lncRNAs in tumors have only been confirmed in lung cancer [20], and there is still a lack of relevant studies in other tumors. This study aims to investigate how lncRNA HEPFAL mediates the occurrence of ferroptosis in HCC.

## Materials and methods

### Cell culture and transfection

HEK293T cells and PLC/PRF/5 cells were purchased from Shanghai Chinese Academy of Sciences (Shanghai, China), and HCCLM3 cells were purchased from the Cell Storage Center of Wuhan University (Wuhan, China). Cells were placed in DMEM (Gibco, #11960-044) at 37 °C in a 5% CO_2_ incubator with 10% fetal bovine serum (Gibco, #10099141) and 1% penicillin and streptomycin (Gibco, #15070-063). Lipofectamine 3000 (Invitrogen, #L3000-015) was used for transfection according to the product instructions.

### Immunohistochemistry and HCC patient samples

The sample was cut into 4 mm sections, hybridized with an anti-SLC7A11 antibody (Proteintech, #26864-1-AP) at 4 °C, and then a DAB chromogenic kit (ZSGB-BIO, #ZLI-9018) was used to detect protein expression. Samples of HCC patients came from the First Affiliated Hospital of Wenzhou Medical University (WMU). All samples were obtained with written informed consent and passed ethical review. To ensure the balance of the number of patients with different stages of HCC and the reliability of the results, the inclusion and exclusion criteria were strictly controlled. The details are as follows. Inclusion criteria are: 1) Accurate pathological diagnosis of primary hepatocellular carcinoma; 2) Obtain complete clinicopathological and follow-up data; 3) Mainly surgical resection; 4) Willing to sign informed consent. The exclusion criteria are (1) perioperative death; (2) undergo palliative surgery; (3) have been treated with chemotherapy drugs; (4) combined with other cancers.

### Western blot analysis and antibodies

The sample was added to a 10% gel for electrophoresis. After electrophoresis, the protein was transferred to the membrane (Millipore, #IPVH00010) and then hybridized with the antibody. Finally, the signal on the membrane was detected by an ECL kit (Thermo, #34096). Antibodies: GPX4 (Abcam, #ab125066), SLC7A11 (Abcam, #ab175186), ACSL4 (Abcam, #ab155282), NRF2 (Proteintech, #16396-1-AP), FSP1 (Proteintech, #20886-1-AP), β-actin (CST, #4970s), PI3K (CST,#4249s), AKT (CST,#9272s), P-AKT (CST,#4060s), and mTOR (CST,#2983s).

### qRT-PCR

TRIzol reagent (Thermo, Ambion) was used to lyse the sample, total RNA was obtained, and a cDNA synthesis kit (Thermo, #K1622) was used to perform reverse transcription according to the product specification. The primer sequences are present in Table S1.

### Measurement of GSH levels, ROS, and Fe^2+^

The GSH-Glo Glutathione Assay Kit (Promega, #V6911) was used to detect the GSH levels according to the product instructions. Dihydroethidium (Beyotime, #S0063) ROS red dye was used as a molecular probe, and FerroOrange (DOJINDO, #F374) was measured with a flow cytometer or observed under a fluorescence microscope.

### Transwell migration and invasion assay

Transwell (Costar, #3422) assays were used to detect cell migration and invasion ability.

### Transmission electron microscopy

A standard procedure was followed using the transmission electron microscope of Wenzhou Medical University.

### Protein degradation assay

MG-132 (MCE, #HY-13259) is a proteasome inhibitor that can effectively inhibit protein degradation. CHX (MCE, #HY-12320) can hinder protein synthesis.

### Construction of lentivirus and xenograft mouse models

HEPFAL overexpression lentiviruses were constructed based on the lentiviral vector pLenti-CMV-GFP-Puro, and the pLenti-CMV-GFP-Puro empty vector was used as control after transfection of cell lines for subsequent experiments. The mouse strain we used was male nude mice, six weeks old. The operation of animals was approved by the Experimental Animal Center of Wenzhou Medical University, which is in line with animal ethics.

### Statistical analysis

Data analysis was performed using GraphPad Prism 8.0 and the R program (version 4.1.3). Continuous variables are presented as the mean ± standard deviation (SD) or median (interquartile range, [IQR]), while categorical variables are described as frequency(percentage). Each experiment was repeated three times. The student’s *t*-test or Wilcoxon rank-sum test was conducted to compare the differences between the two groups for continuous variables. One-way ANOVA or Kruskal–Wallis test was used to determine differences among more than two groups. Chi-square test or Fisher’s exact test were performed for categorical variables. Overall survival (OS) analysis was used the Kaplan–Meier (K–M) method. *P* < 0.05(two-tailed) indicated statistically significant.

## Results

### The expression of HEPFAL is correlated with SLC7A11 in HCC

**Fig. 1:**
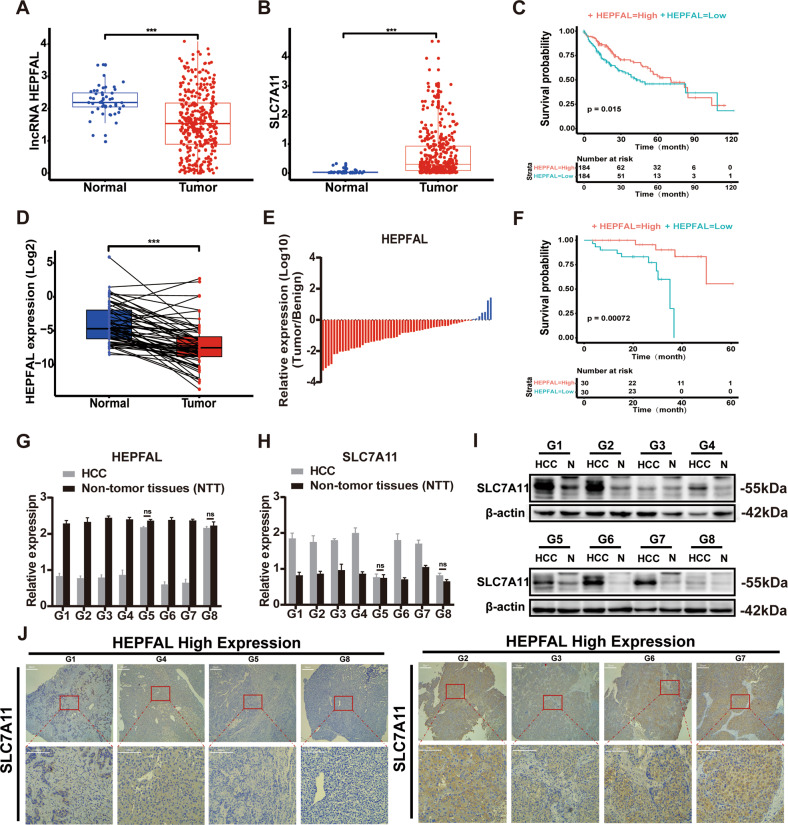
lncRNA HEPFAL is downregulated in HCC, indicating a poor prognosis, and SLC7A11 is up-regulated. **A** The expression of lncRNA HEPFAL in HCC is lower than that in normal tissues (*n* = 368 in TCGA database). **B** The expression of SLC7A11 in HCC is higher than that in normal tissues (*n* = 368 in TCGA database). **C** Based on the median expression of lncRNA HEPFAL, patients were divided into high-expression and low-expression groups, and the Kaplan–Meier survival curve of 368 HCC patients was drawn. **D**, **E** qRT-PCR detects the expression of lncRNA HEPFAL in tumor tissues and matched normal tissues (*n* = 60 in WMU hospital). **F** Kaplan–Meier survival curve of overall survival in 60 HCC patients according to the lncRNA HEPFAL expression. Patients were stratified into high and low expression groups by median expression. **G**, **H** qRT-PCR detects the expression of lncRNA HEPFAL and SLC7A11. **I** The expression of SLC7A11 was detected by western blot. **J** IHC detected SLC7A11 expression in tumor tissues with high and low lncRNA HEPFAL expression. ***P < 0.001.

The lncRNA data were downloaded through the TCGA data portal (https://portal.gdc.cancer.gov/). We performed differential expression analysis on 368 tumor tissues and 50 matched adjacent tissues by DEseq and edgeR R package and obtained 416 differentially expressed lncRNAs in HCC (Fig. S1). Subsequently, we obtained 40 ferroptosis-related genes from MalaCards (http://pathcards.genecards.org/card/ferroptosis) and obtained seven genes through differential expression analysis (Fig. S2). Through prognostic analysis of clinical data, we finally chose SLC7A11. Then, we analyzed the correlation between 416 lncRNAs and SLC7A11, and we obtained lncRNA KB-68A7.1 (ENSG00000274225.1), which is most significantly related to the key ferroptosis protein SLC7A11 in HCC. For this reason, we named it lncRNA HEPFAL (hepatocellular carcinoma ferroptosis associative lncRNA). At the same time, we analyzed the ferroptosis key protein SLC7A11 in the TCGA database and found that SLC7A11 is highly expressed in cancer tissues, and its expression is related to the prognosis of patients (Fig. S3). We found that the expression of lncRNA HEPFAL in tumor tissues was significantly lower than that in normal tissues, while SLC7A11 expression was higher in tumor tissues (Fig. 1A, B). Further, we ascertained the relationships between the expression levels of lncRNA HEPFAL and the clinicopathologic characteristics of HCC patients (Table 1). Through K–M analysis of HCC patients in TCGA, we found that the OS of patients with high expression of lncRNA HEPAL was longer than that of patients with low expression (Fig. 1C), which was consistent with the expression trend of lncRNA HEPFAL in tumor and normal tissues.

**Table 1:** Association of lncRNA HEPFAL expression and clinicopathological features of HCC patients in TCGA database (*n* = 368).

Characteristics total		HEPFAL Expression		*P*-value
		Low (*n* = 184)	High (*n* = 184)	
Gender				0.396
Female	119 (32.34)	64 (34.78)	55 (29.89)	
Male	249 (67.66)	120 (65.22)	129 (70.11)	
Age(year)				0.535
<=60	175 (47.55)	91 (49.46)	84 (45.65)	
>60	193 (52.45)	93 (50.54)	100 (54.35)	
BMI				0.302
<18.5	21 (6.29)	13 (7.93)	8 (4.71)	
18.5-24.9	157 (47.0)	80 (48.78)	77 (45.29)	
>=25	156 (46.7)	71 (43.29)	85 (50.00)	
NA	34			
Adjacent Inflammation				0.031
None	117 (50.21)	68 (57.14)	49 (42.98)	
Mild	99 (42.49)	44 (36.98)	55 (48.25)	
Severe	17 (7.30)	7 (5.88)	10 (8.77)	
NA	135			
T Stage				0.001
T1	181 (49.4)	75 (41.21)	106 (57.61)	
T2	92 (25.14)	50 (27.47)	42 (22.83)	
T3	80 (21.86)	50 (27.47)	30 (16.30)	
T4	13 (3.55)	7 (3.85)	6 (3.26)	
NA	2			
Lymph Node Stage				1.000
N0	252 (98.4)	130 (98.48)	122 (98.39)	
N1	4 (1.56)	2 (1.52)	2 (1.61)	
NA	112			
M Stage				
M0	266 (98.5)	133 (98.52)	133 (98.52)	
M1	4 (1.48)	2 (1.48)	2 (1.48)	
NA	98			
TNM Stage				0.001
Stage I	171 (49.42)	72 (41.38)	99 (57.56)	
Stage II	85 (24.57)	44 (25.29)	41 (23.84)	
Stage III	85 (24.57)	55 (31.61)	30 (17.44)	
Stage IV	5 (1.44)	3 (1.72)	2 (1.16)	
NA	22			
Histologic Grade				<0.0001
G1	55 (15.07)	16 (8.79)	39 (21.31)	
G2	177 (48.49)	85 (46.70)	92 (50.27)	
G3	121 (33.15)	72 (39.56)	49 (25.78)	
G4	12 (3.29)	9 (4.95)	3 (1.64)	
NA	3			
Vascular Invasion				0.741
None	206 (65.60)	104 (67.53)	102 (63.75)	
Micro	92 (29.30)	42 (27.27)	50 (31.25)	
Macro	16 (5.10)	8 (5.20)	8 (5.00)	
NA	54			
Family History of Cancer				0.650
NO	207 (64.89)	109 (66.06)	98 (63.64)	
YES	112 (35.11)	56 (33.94)	56 (36.36)	
NA	49			
History of Risk Factor				0.433
Alcohol Consumption	68 (35.60)	36 (38.72)	32 (32.65)	
Hemochromatosis	5 (2.62)	4 (4.30)	1 (1.02)	
Hepatitis B	75 (39.27)	33 (35.48)	42 (42.86)	
Hepatitis C	32 (16.76)	16 (17.20)	16 (16.33)	
NAFLD	11 (5.75)	4 (4.30)	7 (7.14)	
NA	177			
Ablation Embolization				0.491
NO	232 (94.69)	112 (95.73)	120 (93.75)	
YES	13 (5.31)	5 (4.27)	8 (6.25)	
NA	123			
Postoperative Radiotherapy				0.829
NO	222 (93.67)	110 (94.02)	112 (93.33)	
YES	15 (6.33)	7 (5.98)	8 (6.67)	
NA	131			
Recurrence				0.871
Extrahepatic Recurrence	42 (23.87)	19 (22.35)	23 (25.28)	
Locoregional Recurrence	52 (29.54)	27 (31.76)	25 (27.47)	
Intrahepatic Recurrence	72 (40.91)	35 (41.18)	37 (40.66)	
New Primary Tumor	10 (5.68)	4 (4.71)	6 (6.59)	
NA	192			
Cancer status				0.380
With tumor	150 (42.74)	78 (45.09)	72 (40.45)	
Tumor free	201 (57.26)	95 (54.91)	106 (59.55)	
NA	17			
SLC7A11 Expression	368	0.460 (1.209)	0.145 (0.332)	0.001

Values are presented as number (%) or median (IQR).

*NA* not available, *NAFLD* non-alcohol fatty liver disease.

To further clarify the role of lncRNA HEPFAL and SLC7A11 in HCC, we collected tumor and normal tissue samples from 60 HCC patients at WMU hospital. The expression of lncRNA HEPFAL was determined by real-time PCR, and the results were consistent with the TCGA database (Fig. 1D, E). According to K–M analysis, the OS of patients with high expression of lncRNA HEPFAL was longer than that of patients with low expression (Fig. 1F).

Then, we selected eight matched samples to detect the expression of SLC7A11 by real-time PCR and western blot, and the results showed that the samples with high expression of lncRNA HEPFAL had lower expression of SLC7A11 (Fig. 1G–I, Fig. S8). We also confirmed this by immunohistochemistry (IHC) of paired tissues (Fig. 1J). The above results indicate that the expression of SLC7A11 in HCC is related to lncRNA HEPFAL.

### The overexpression of HEPFAL decreases SLC7A11 expression and inhibits tumor proliferation and migration ability

**Fig. 2:**
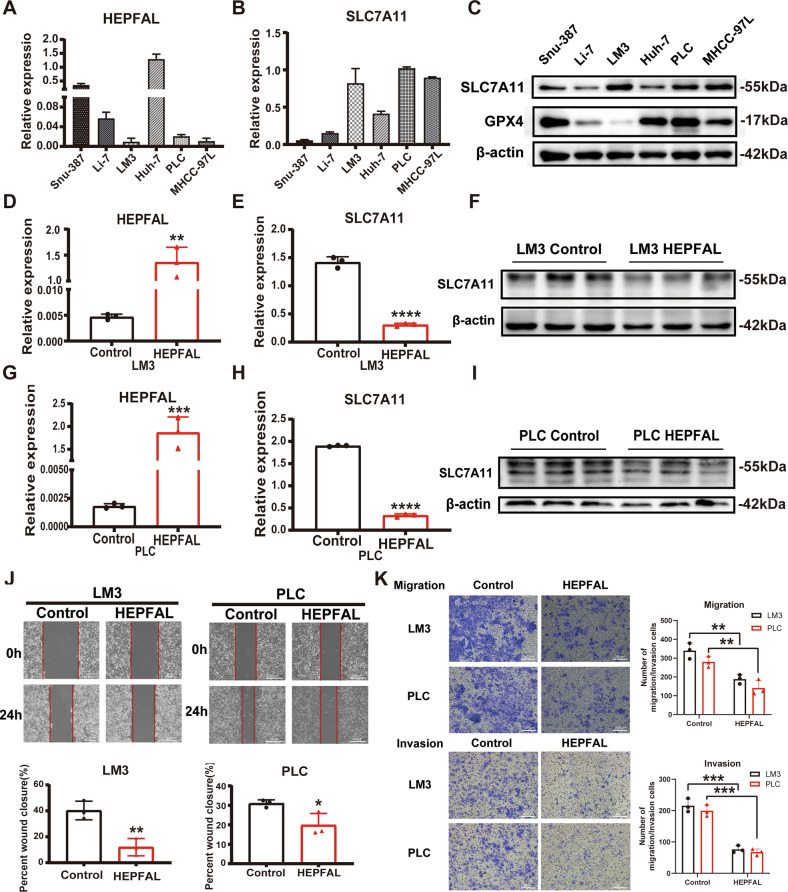
Overexpression of lncRNA HEPFAL inhibits cell colony formation, migration, and invasion in vitro. **A**, **B** Relative expression of lncRNA HEPFAL and SLC7A11 in 6 different liver cancer cell lines was detected by qRT-PCR. **C** The expression of SLC7A11 in 6 different liver cancer cell lines was detected by western blot. **D**, **E**, **G**, **H** Relative expression of lncRNA HEPFAL and SLC7A11 in HCCLM3 and PLC cells transfected with lncRNA HEPFAL expression vector and control vector, respectively. **F**, **I** The expression of SLC7A11 and GPX4 in HCCLM3 and PLC cells transfected with lncRNA HEPFAL expression vector and control vector were detected by western blot. **J** Cell migration capacities were conducted by wound healing assays after transfection. **K** Cell migration and invasion abilities were indicated by transwell assays in each group. *P < 0.05, **P < 0.01, ***P < 0.001, ****P < 0.0001.

To verify the regulation of lncRNA HEPFAL on SLC7A11, we detected the expression levels of lncRNA HEPFAL and SLC7A11 in liver cancer cell lines (Fig. 2A–C, Fig. S9). Based on the above results, we chose LM3 and PLC cells for further research. To reveal the biological effects of HEPFAL, we used lentiviral vectors to stably overexpress HEPFAL in LM3 and PLC cells and then tested the efficiency of overexpression (Fig. 2D–H, Fig. S4). We found that after overexpression of HEPFAL, the expression of SLC7A11 in cells decreased (Fig. 2F, I, Fig. S10, 11). In addition, we performed wound healing experiments and transwell assays. The results showed that cells have strong migration and invasion abilities in the case of empty vectors (Fig. 2K). Wound healing experiments also showed the same result (Fig. 2J). The number of colonies of LM3 and PLC cells stably overexpressing lncRNA HEPFAL was less than that of the control group (Fig. S6**)**. The above results indicate that HEPFAL may regulate ferroptosis through SLC7A11 and inhibit tumor proliferation and migration.

### The overexpression of HEPFAL promotes ferroptosis

**Fig. 3:**
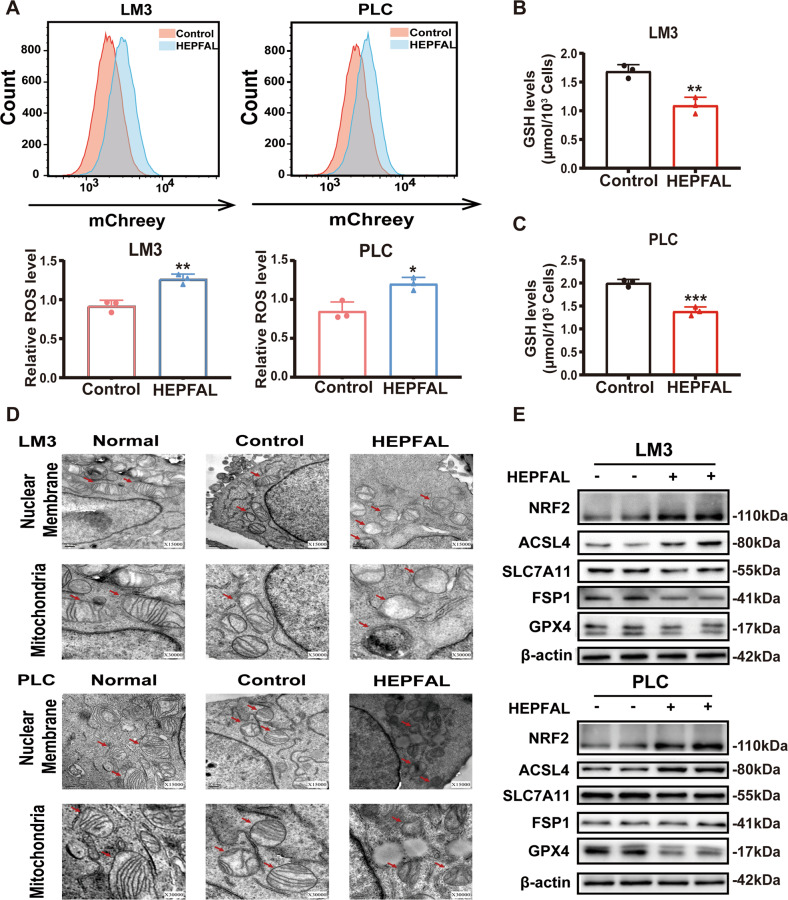
Overexpression of lncRNA HEPFAL promotes cell ferroptosis. **A** FACS and statistical analysis of ROS level in HCCLM3 and PLC cells stably overexpressing lncRNA HEPFAL. **B**, **C** Intracellular GSH levels were examined in HCCLM3 and PLC cells treated as indicated, and bar graphs are shown. **D** HCCLM3 and PLC cells stably overexpressing lncRNA HEPFAL were subjected to transmission electron microscopy. Representative images are shown. **E** Western blot analysis of NRF2, ACSL4, SLC7A11, FSP1 and GPX4 protein level in HCCLM3 and PLC cells transfected with lncRNA HEPFAL expression vector and control vector. *P < 0.05, **P < 0.01, ***P < 0.001.

As mentioned earlier, HEPFAL may induce changes in the tumor phenotype through ferroptosis. The accumulation of lipid ROS in cells is one of the characteristic manifestations of ferroptosis [21]. After overexpression of HEPFAL, we used a superoxide anion fluorescent probe and found that HEPFAL increased the total ROS level in cells (Fig. 3A). The uptake of extracellular cystine is mainly mediated by SLC7A11, and cystine is used as a synthetic raw material for glutathione (GSH), a reducing substance in cells [22]. Therefore, we tested the GSH level in the cells and found that after transfection of the HEPFAL overexpression vector, the level of GSH in the cells decreased (Fig. 3B, C). We observed that the cells overexpressing HEPFAL showed changes in mitochondrial morphology compared to the empty vector and normal cells, including smaller size, the disappearance of mitochondrial cristae, and increased density of mitochondrial membrane, and the nuclear membrane was still intact (Fig. 3D). Meanwhile, the western blot results showed that the levels of the ferroptosis inhibitors SLC7A11 and GPX4 were decreased in cells stably overexpressing HEPFAL. Ferroptosis promoters, including acyl-coenzyme A synthetase long-chain family member 4 (ACSL4) and nuclear factor erythrid-2 related factor (NRF2), were increased due to overexpression of HEPFAL, and the change in ferroptosis suppressor protein 1 (FSP1) was not obvious (Fig. 3E, Fig. S12). These results indicate that the overexpression of HEPFAL impairs the activity of SLC7A11 in the cell and leads to the accumulation of lipid ROS, ultimately leading to ferroptosis.

### The overexpression of HEPFAL makes cells susceptible to ferroptosis

**Fig. 4:**
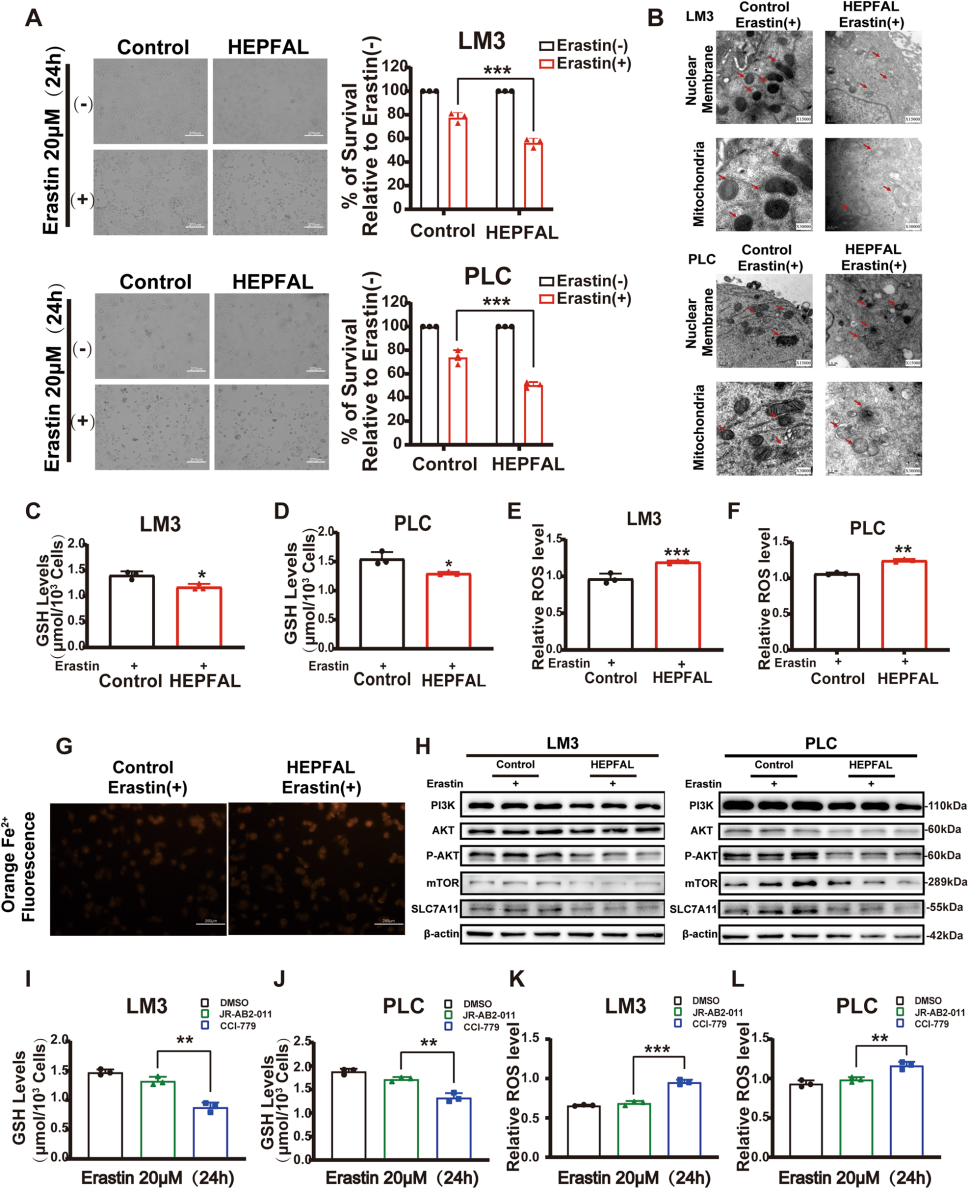
Overexpression of lncRNA HEPFAL increases the sensitivity of erastin-induced ferroptosis. **A** HCCLM3 and PLC cells transfected with lncRNA HEPFAL expression vector or control vector were treated with 20 μM erastin or untreated for 24 h and counted the surviving cells. **B** HCCLM3 and PLC cells were transfected with lncRNA HEPFAL expression vector and control vector treated with 20 μM erastin and were subjected to transmission electron microscopy. Representative images are shown. **C**, **D** Intracellular GSH levels were examined in HCCLM3 and PLC cells treated as (**A**). **E**, **F** FACS detected ROS levels in HCCLM3 and PLC cells treated as (**A**). **G** HCCLM3 cells stably overexpressing lncRNA HEPFAL were treated with 20 μM erastin to visualize the intracellular Fe^2+^ ions generation using the Fe^2+^ ions fluorescent probe Ferrorange. **H** Western blot analysis of PI3K, AKT, P-AKT, mTOR, SLC7A11, and GPX4 protein level in HCCLM3 and PLC cells transfected with lncRNA HEPFAL expression vector and control vector treated with 20 μM erastin. **I**, **J** Intracellular GSH levels were examined in HCCLM3 and PLC cells treated with DMSO, JR-AB2-011, and CCI-779. **K**, **L** FACS detected ROS levels in HCCLM3 and PLC cells treated as (**I**, **J**). *P < 0.05, **P < 0.01, ***P < 0.001.

Erastin is a small molecule compound that can induce ferroptosis, mainly through system Xc- [23]. After treating cells with erastin, we found cell death in both groups, but the survival rate of cells overexpressing HEPFAL was significantly lower than that of empty vector (Fig. 4A). Subsequently, we detected the level of GSH in the cell. It is worth noting that the glutathione level in the cells was lower in the cells overexpressing HEPFAL (Fig. 4C, D). We also tested the total intracellular ROS levels. The results show that the overexpression of HEPFAL increased the ROS levels induced by erastin (Fig. 4E, F). The process of cell ferroptosis is accompanied by the accumulation of many iron ions. Fe^3+^ enters the cell through TfR and is reduced to Fe^2+^ and stored in ferritin. Excess iron ions can generate ROS through the Fenton reaction [24]. In LM3 cells overexpressing HEPFAL, Fe^2+^ presented a higher level (Fig. 4G). This shows that after overexpression of HEPFAL, cells are more prone to ferroptosis. Transmission electron microscope also showed the same result (Fig. 4B). Recently, a study showed that inhibiting PI3K-AKT-mTOR signaling can increase the ferroptosis sensitivity of RSL3 to cancer cells [25]. Therefore, we detected the expression level of the PI3K-AKT-mTOR signaling pathway. The results showed that the PI3K-AKT-mTOR signaling pathway was inhibited after the cells overexpressing HEPFAL were treated with erastin (Fig. 4H, Fig. S13), which indicates that lncRNA HEPFAL increases sensitivity to erastin-induced ferroptosis and may be related to the PI3K-AKT-mTOR signaling pathway. To determine the complexes associated with ferroptosis, we tested the effects of cells on erastin-induced ferroptosis by CCI-779 (mTORC1 inhibitor) and JR-AB2-011 (mTORC2 inhibitor). The results showed that the ferroptosis of cells treated with CCI-779 was more serious than that of the control group and JR-AB2-011 group (Fig. S5). The level of GSH content and the level of total intracellular ROS shows the same results (Fig. 4I–L). The above results suggest that the increased sensitivity to erastin-induced ferroptosis caused by lncRNA HEPFAL may be mediated by mTORC1.

### HEPFAL regulates ferroptosis by SLC7A11

**Fig. 5:**
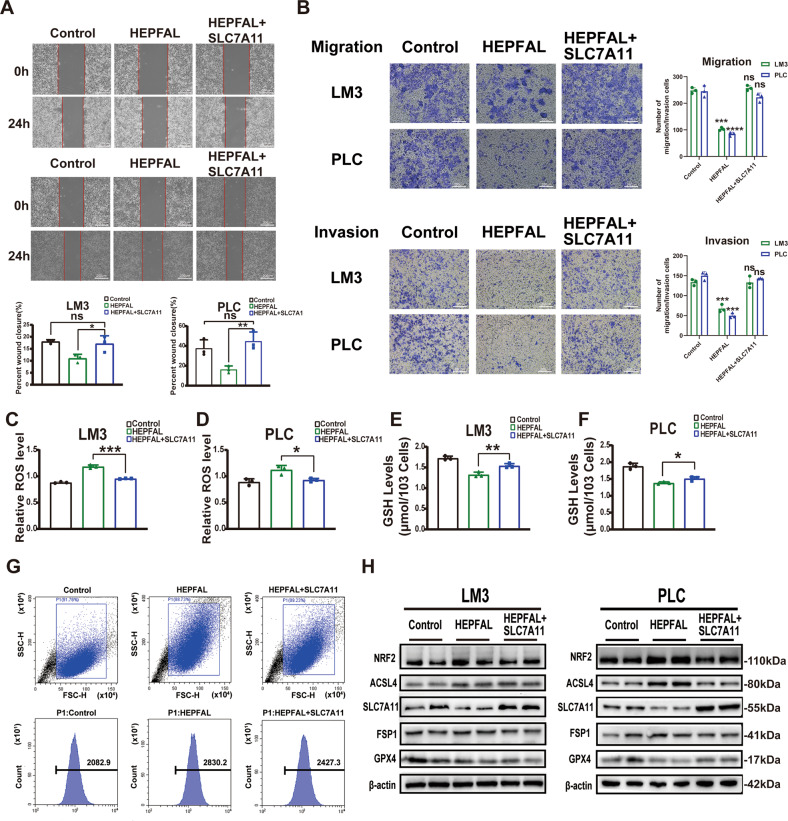
SLC7A11 rescued ferroptosis caused by the overexpression of lncRNA HEPFAL. **A** HCCLM3 and PLC cell migration capacities were conducted by wound healing assays after transfected. **B** Cell migration and invasion abilities were indicated by transwell assays in each group. **C**, **D** ROS level was detected by FACS in HCCLM3 and PLC cells transfected as indicated. **E**, **F** Intracellular GSH levels were examined in HCCLM3 and PLC cells transfected as indicated. **G** FACS assessed intracellular Fe^2+^ ions generation in HCCLM3 and PLC cells transfected as indicated. **H** Western blot analysis of NRF2, ACSL4, SLC7A11, FSP1, and GPX4 protein levels in HCCLM3 and PLC cells transfected as indicated. ns is not significant, *P < 0.05, **P < 0.01, ***P < 0.001, ****P < 0.0001.

As mentioned earlier, we found that overexpression of HEPFAL downregulated the gene and protein levels of SLC7A11. Therefore, we transfected the vector overexpressing SLC7A11 into cells stably overexpressing HEPFAL. Consistent with the previous results, the migration and invasion ability of cells overexpressing HEPFAL decreased, while the migration and invasion ability of cells overexpressing HEPFAL and SLC7A11 was significantly restored (Fig. 5B). The same result was also shown in the wound healing experiment (Fig. 5A). In addition, the cells overexpressing HEPFAL and SLC7A11 demonstrated an increased number of colonies (Fig. S6). The results of total intracellular ROS levels (Fig. 5C, D) and GSH levels (Fig. 5E, F) also showed the same trend. Moreover, in the cells overexpressing HEPFAL and SLC7A11, the Fe^2+^ level was reduced to a certain extent (Fig. 5G). Western blot analysis showed that after overexpression of HEPFAL and SLC7A11, the expression of NRF2 and ACSL4 increased, while the expression of GPX4 decreased, and the changing trend of FSP1 was not obvious (Fig. 5H, Fig. S14). The above results indicate that the ferroptosis caused by overexpression of HEPFAL is mediated by SLC7A11.

### HEPFAL regulates SLC7A11 through ubiquitination

**Fig. 6:**
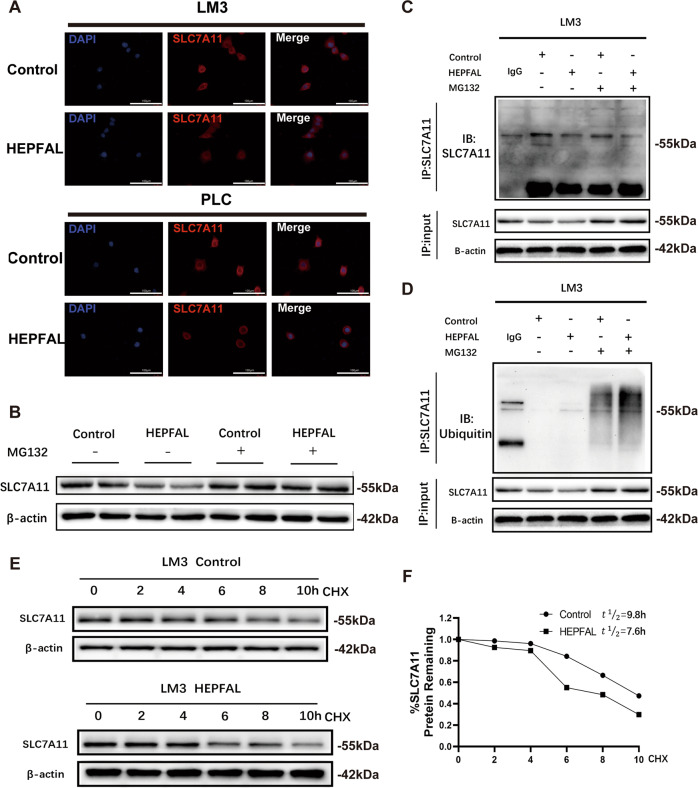
Overexpression of lncRNA HEPFAL decreased the stability of SLC7A11 protein. **A** SLC7A11 immunofluorescence in HCCLM3 and PLC cells transfected as indicated. **B** Western blot analysis of SLC7A11 in HCCLM3 cells after transfected with lncRNA HEPFAL expression vector or control vector and treated with 20 μM MG-132 or untreated for 8 h. **C, D** LncRNA HEPFAL expression vector or control vector was transfected into HCCLM3 cells. After the cells were treated with or without 20 μM MG-132 for 8 h, SLC7A11 was immunoprecipitated with anti-SLC7A11 antibody, and the ubiquitination of SLC7A11 was examined by western blot. **E**, **F** HCCLM3 cells transfected with lncRNA HEPFAL expression vector or control vector were treated with 50 mg/ml CHX and detected the expression of SLC7A11 by western blot. The intensity of endogenous SLC7A11 expression for each time point was quantified by densitometry and drawn into a line chart.

To explore the specific mechanism by which HEPFAL regulates SLC7A11, we used SLC7A11 immunofluorescence and found that SLC7A11 is localized in the cytoplasm. Consistent with previous results, SLC7A11 expression was lower in cells overexpressing HEPFAL (Fig. 6A). We predicted the subcellular location of HEPFAL through the lncLocator website (http://www.csbio.sjtu.edu.cn/bioinf/lncLocator/) and found that the predicted location of HEPFAL was in the cytoplasm (Fig. S7). LncRNAs regulate protein levels by interacting with proteins [26]. Then we detected by western blot that the expression of SLC7A11 was reduced in cells overexpressing HEPFAL that were not treated with MG-132, and interestingly, in the overexpressing HEPFAL cells treated with MG-132, the expression of SLC7A11 returned to the same level as that in normal cells (Fig. 6B, Fig. S15). This interesting result suggests that HEPFAL may modulate the expression of SLC7A11 through ubiquitination. To further verify this conjecture, we performed an immunoprecipitation experiment to extract the endogenous SLC7A11 protein from cells (Fig. 6C, Fig. S16) and detected the ubiquitination of the SLC7A11 protein (Fig. 6D, Fig. S17). We found that in cells overexpressing HEPFAL, the ubiquitination of endogenous SLC7A11 protein increased. This proves our previous conjecture that overexpression of HEPFAL resulted in a decrease in the expression of SLC7A11, which may be caused by ubiquitination modification. Then, we measured the half-life of the SLC7A11 protein (Fig. 6E, F, Fig. S18) and found that the half-life (7.6 h) of SLC7A11 protein-overexpressing HEPFAL cells was lower than that of normal cells (9.8 h). In summary, these findings indicate that HEPFAL regulates SLC7A11 through ubiquitination modification, which affects the stability of the SLC7A11 protein. However, its specific mechanism of action is still unclear.

### HEPFAL inhibits tumor growth of cell xenografts in nude mice

**Fig. 7:**
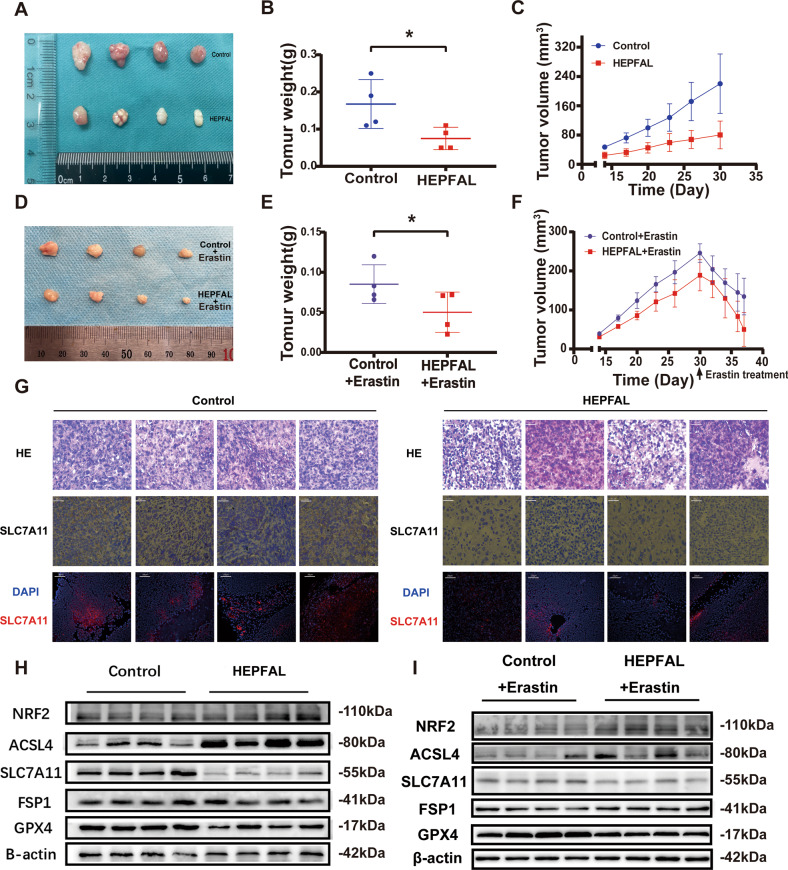
Overexpression of lncRNA HEPFAL inhibits tumor growth of cell xenografts in nude mice. **A** 1 × 10^6^ HCCLM3 cells were injected into nude mice and removed after 30 days. **B** Measure and count the weight of the tumor(*n* = 4). **C** The tumor volumes were measured every three days after the cells were injected for 14 days. **D** HCCLM3 cells were injected into nude mice and treated with erastin after 30 days. **E** Measure and count the weight of the tumor (*n* = 4). **F** The tumor volumes were measured every three days after the cells were injected for 14 days. **G** SLC7A11 expression was detected by IHC and IF. **H** Western blot analysis of NRF2, ACSL4, SLC7A11, FSP1, and GPX4 protein level in tumor tissues. **I** Western blot analysis of NRF2, ACSL4, SLC7A11, FSP1, and GPX4 protein levels in tumor tissues after erastin treatment. *P < 0.05.

The tumor volume and weight of the HEPFAL overexpression group were significantly reduced (Fig. 7A–C). In addition, we found that the expression level of SLC7A11 in the overexpressing HEPFAL group was lower than that of the control group, and the number of tumor cells in the overexpression HEPFAL group was the lowest. In contrast, the number of tumor cells in the control group was higher (Fig. 7G). As an essential part of ferroptosis research, erastin treated the mouse models of subcutaneous tumors in the control and HEPFAL group. The results showed that the mice in the control group and the HEPFAL overexpression group both had therapeutic effects on the tumor (Fig. 7D, E). However, the tumor treatment effect of the group of HEPFAL overexpression was more significant (Fig. 7F), which also verified our previous in vivo experiments that HEPFAL can increase the sensitivity of erastin-induced ferroptosis. The western blot results showed that compared with the control group, the expression levels of SLC7A11 and GPX4 decreased, while the expression levels of NRF2 and ACSL4 increased, and there was no significant change in FSP1 (Fig. 7H, I, Fig. S19, 20). These results are consistent with the above in vitro experimental results.

## Discussion

LncRNAs have a dynamic role in the process of transcription and translation and are closely related to cancer progression [27]. This study reported that lncRNA HEPFAL, upstream of the key ferroptosis gene SLC7A11, regulates ferroptosis in liver cancer cells. We found in the TCGA database that lncRNA HEPFAL is significantly lower in HCC tissues than in normal tissues. Through the analysis of differences, we found that it is significantly related to cell ferroptosis. Therefore, we collected 60 patient samples with cancer and normal tissues and found that the expression of lncRNA HEPFAL in HCC tissues was lower than that in normal liver tissues, which was in line with the trend in the TCGA database. In addition, in vitro and in vivo experiments verified that lncRNA HEPFAL reduces the migration and invasion of liver cancer cells and promotes ferroptosis of liver cancer cells. This indicates that lncRNA HEPFAL is directly related to ferroptosis and its molecular mechanism. Recent studies have found that ferroptosis suppressor protein (FSP1) plays a role in ferroptosis and that FSP1 can inhibit ferroptosis by reducing CoQ [28, 29]. However, in our study, the expression of FSP1 did not appear to have changed significantly. This may be because FSP1 inhibits ferroptosis in parallel with the SLC7A11-GPX4 axis, and the lncRNA HEPFAL may mainly act on SLC7A11. The changes in NRF2, ACSL4, and GPX4 revealed the influence of lncRNAs on the ferroptosis process. In addition, we found that lncRNA HEPFAL can increase the sensitivity of cells to erastin-induced ferroptosis. Erastin is a ferroptosis inducer that mainly acts on system Xc-, and this result further explains the regulatory effect of lncRNA HEPFAL on SLC7A11. In normal cells, system Xc- acts as a cystine/glutamate transporter, transporting a cysteine molecule into the cell to exchange for a glutamate molecule in the cell [30]. SLC7A11 is an important part of system Xc-. Cystine taken up into cells by SLC7A11 is converted into cysteine by a reduction reaction that consumes NADPH, and cysteine is a synthetic raw material for GSH [31, 32]. Our study found that lncRNA HEPFAL acts on SLC7A11 and reduces its expression, which leads to a decrease in the amount of cystine transported into the cell, resulting in a decrease in the level of GSH in the cell and thereby effectively inhibiting the ferroptotic function of GPX4 and eventually leading to the ferroptosis of tumor cells. In addition, we found that the PI3K-AKT-mTOR signaling pathway plays a role in this process. We believe that PI3K can activate AKT through downstream molecules and that AKT can activate mTOR through phosphorylation. mTOR contains two complexes, mTORC1 and mTORC2. mTORC1 activated by AKT mainly regulates various molecules, such as SLC7A11, GPX4 and ferroportin, through NRF2 to regulate ferroptosis. We found that erastin-induced ferroptosis may occur mainly through mTORC1, which may be related to the effect of mTORC1 on SLC7A11 and GPX4. Based on the above experimental results, we speculate that lncRNA HEPFAL activates mTORC1 through the PI3K-AKT-mTOR signaling pathway to act on SLC7A11, increasing the sensitivity to erastin-induced ferroptosis. The specific mechanism needs further experimental verification. Recent studies have pointed out that P53 can promote ferroptosis by transcriptionally inhibiting the expression of SLC7A11. Further research showed that P53 might suppress the expression of SLC7A11 by downregulating the H2Bub1 level of its gene regulatory region. These studies indicate that the regulation of SLC7A11 may be related to modifications such as methylation, acetylation, and ubiquitination [16, 33, 34]. In addition, studies have pointed out that P53-mediated ferroptosis is related to lncRNAs [19]. LncRNAs exert their effects by interacting with proteins [35–37]. In this study, we found that the lncRNA HEPFAL was localized in the cytoplasm and decreased in the expression level of the transmembrane protein SLC7A11. Therefore, we speculated that SLC7A11 might undergo protein degradation at the translation level. The expression level of endogenous SLC7A11 in cells was detected, and it was found that there was no difference in the expression level of SLC7A11 after the application of the proteasome inhibitor MG-132, while the ubiquitination site was detected. The protein level of SLC7A11 was significantly increased, which indicated that SLC7A11 was probably degraded by ubiquitination under the action of lncRNA. After applying CHX, we also verified that the stability of SLC7A11 protein in cells overexpressing lncRNA HEPFAL decreased and was more prone to degradation, indicating that lncRNA HEPFAL may be degraded by ubiquitination-mediated SLC7A11 protein. The specific molecular mechanism needs to be further studied. In addition, some lncRNAs have also been shown to be independent biomarkers for the prognosis of HCC patients, such as lncRNA PICSAR, LINC01093, and lncRNA H19 [38–40]. Our above experiments show that lncRNA HEPFAL is closely related to the occurrence and development of ferroptosis in HCC and affects the occurrence and development of tumors. Therefore, we believe that with the further deepening of molecular mechanisms, lncRNA HEPFAL has great potential as a therapeutic target.

**Fig. 8:**
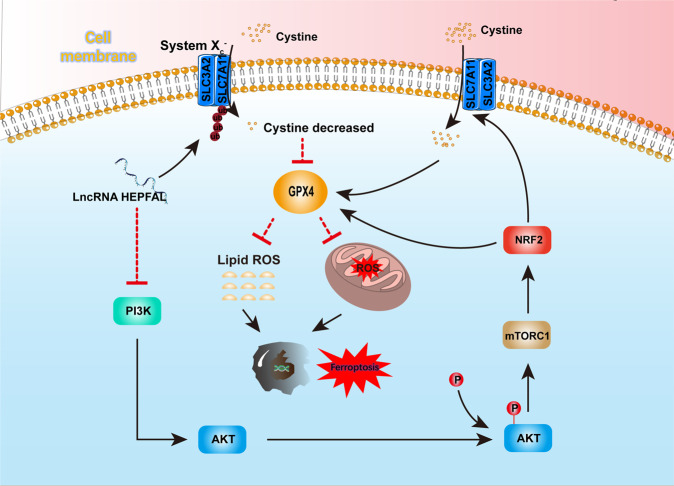
LncRNA HEPFAl promotes ferroptosis by modifying SLC7A11 ubiquitination. Working model for lncRNA HEPFAL in cell ferroptosis.

In general, our research found that lncRNA HEPFAL promotes the ubiquitination of SLC7A11, resulting in a decrease in GSH production, which in turn affects the activity of GPX4 and ultimately leads to the occurrence of ferroptosis (Fig. 8). We discovered a novel lncRNA and verified its significant correlation with patient prognosis in hepatocellular carcinoma. It also explained that the lncRNA HEPFAL mediates ferroptosis in hepatoma cells by regulating the ferroptosis key protein SLC7A11 through ubiquitination modification. This provides a new understanding of hepatocellular carcinoma’s occurrence and development mechanism. LncRNA HEPFAL has the potential as a target for the diagnosis and treatment of HCC.

## Availability of data and materials

The data used to support the findings of this study are available from the corresponding author upon request.

## Supplementary information

Supplementary information

original western blots

Reproducibility Checklist

## References

[1] Akinyemiju T, Abera S, Ahmed M, Alam N, Alemayohu MA, Global Burden of Disease Liver Cancer Collaboration (2017). The Burden of Primary Liver Cancer and Underlying Etiologies From 1990 to 2015 at the Global, Regional, and National Level: Results From the Global Burden of Disease Study 2015. JAMA Oncol.

[2] Yang JD, Hainaut P, Gores GJ, Amadou A, Plymoth A, Roberts LR (2019). A global view of hepatocellular carcinoma: trends, risk, prevention and management. Nat Rev Gastroenterol Hepatol.

[3] Craig AJ, von Felden J, Garcia-Lezana T, Sarcognato S, Villanueva A (2020). Tumour evolution in hepatocellular carcinoma. Nat Rev Gastroenterol Hepatol.

[4] Khalil AM, Guttman M, Huarte M, Garber M, Raj A, Rivea Morales D (2009). Many human large intergenic noncoding RNAs associate with chromatin-modifying complexes and affect gene expression. Proc Natl Acad Sci USA.

[5] Bhan A, Soleimani M, Mandal SS (2017). Long noncoding RNA and cancer: a new paradigm. Cancer Res.

[6] Huang Z, Zhou JK, Peng Y, He W, Huang C (2020). The role of long noncoding RNAs in hepatocellular carcinoma. Mol cancer.

[7] Dixon SJ, Lemberg KM, Lamprecht MR, Skouta R, Zaitsev EM, Gleason CE (2012). Ferroptosis: an iron-dependent form of nonapoptotic cell death. Cell.

[8] Koppula P, Zhuang L, Gan B (2021). Cystine transporter SLC7A11/xCT in cancer: ferroptosis, nutrient dependency, and cancer therapy. Protein cell.

[9] Hassannia B, Vandenabeele P, Vanden Berghe T (2019). Targeting ferroptosis to iron out cancer. Cancer cell.

[10] Tang D, Kang R, Berghe TV, Vandenabeele P, Kroemer G (2019). The molecular machinery of regulated cell death. Cell Res.

[11] Friedmann Angeli JP, Krysko DV, Conrad M (2019). Ferroptosis at the crossroads of cancer-acquired drug resistance and immune evasion. Nat Rev Cancer.

[12] Stockwell BR, Friedmann Angeli JP, Bayir H, Bush AI, Conrad M, Dixon SJ (2017). Ferroptosis: a regulated cell death nexus linking metabolism, redox biology, and disease. Cell.

[13] Xie Y, Hou W, Song X, Yu Y, Huang J, Sun X (2016). Ferroptosis: process and function. Cell Death Differ.

[14] Chen D, Fan Z, Rauh M, Buchfelder M, Eyupoglu IY, Savaskan N (2017). ATF4 promotes angiogenesis and neuronal cell death and confers ferroptosis in a xCT-dependent manner. Oncogene.

[15] Tang B, Zhu J, Li J, Fan K, Gao Y, Cheng S (2020). The ferroptosis and iron-metabolism signature robustly predicts clinical diagnosis, prognosis and immune microenvironment for hepatocellular carcinoma. Cell Commun Signal.

[16] Jiang L, Kon N, Li T, Wang SJ, Su T, Hibshoosh H (2015). Ferroptosis as a p53-mediated activity during tumour suppression. Nature.

[17] Wang M, Mao C, Ouyang L, Liu Y, Lai W, Liu N (2019). Long noncoding RNA LINC00336 inhibits ferroptosis in lung cancer by functioning as a competing endogenous RNA. Cell Death Differ.

[18] Wang Z, Chen X, Liu N, Shi Y, Liu Y, Ouyang L (2021). A nuclear long non-coding RNA LINC00618 accelerates ferroptosis in a manner dependent upon apoptosis. Mol Ther: J Am Soc Gene Ther.

[19] Mao C, Wang X, Liu Y, Wang M, Yan B, Jiang Y (2018). A G3BP1-interacting lncRNA promotes ferroptosis and apoptosis in cancer via nuclear sequestration of p53. Cancer Res.

[20] Wu Y, Zhang S, Gong X, Tam S, Xiao D, Liu S (2020). The epigenetic regulators and metabolic changes in ferroptosis-associated cancer progression. Mol Cancer.

[21] Ma S, Dielschneider RF, Henson ES, Xiao W, Choquette TR, Blankstein AR (2017). Ferroptosis and autophagy induced cell death occur independently after siramesine and lapatinib treatment in breast cancer cells. PLoS ONE.

[22] Polewski MD, Reveron-Thornton RF, Cherryholmes GA, Marinov GK, Aboody KS (2017). SLC7A11 overexpression in glioblastoma is associated with increased cancer stem cell-like properties. Stem Cells Dev.

[23] Oh BM, Lee SJ, Park GL, Hwang YS, Lim J, Park ES (2019). Erastin inhibits septic shock and inflammatory gene expression via suppression of the NF-κB pathway. J Clin Med.

[24] Zhao Y, Li J, Guo W, Li H, Lei L (2020). Periodontitis-level butyrate-induced ferroptosis in periodontal ligament fibroblasts by activation of ferritinophagy. Cell Death Discov.

[25] Yi J, Zhu J, Wu J, Thompson CB, Jiang X (2020). Oncogenic activation of PI3K-AKT-mTOR signaling suppresses ferroptosis via SREBP-mediated lipogenesis. Proc Natl Acad Sci USA.

[26] Zhang W, Yue X, Tang G, Wu W, Huang F, Zhang X (2018). SFPEL-LPI: Sequence-based feature projection ensemble learning for predicting LncRNA-protein interactions. PLoS Comput. Biol.

[27] Wang K, Liu F, Zhou LY, Long B, Yuan SM, Wang Y (2014). The long noncoding RNA CHRF regulates cardiac hypertrophy by targeting miR-489. Circulation Res.

[28] Bersuker K, Hendricks JM, Li Z, Magtanong L, Ford B, Tang PH (2019). The CoQ oxidoreductase FSP1 acts parallel to GPX4 to inhibit ferroptosis. Nature.

[29] Doll S, Freitas FP, Shah R, Aldrovandi M, da Silva MC, Ingold I (2019). FSP1 is a glutathione-independent ferroptosis suppressor. Nature.

[30] He Y, Hewett SJ (2022). The cystine/glutamate antiporter, system x_c_^-^, contributes to cortical infarction after moderate but not severe focal cerebral ischemia in mice. Front Cell Neurosci.

[31] Dixon SJ, Patel DN, Welsch M, Skouta R, Lee ED, Hayano M (2014). Pharmacological inhibition of cystine-glutamate exchange induces endoplasmic reticulum stress and ferroptosis. eLife.

[32] Muir A, Danai LV, Gui DY, Waingarten CY, Lewis CA, Vander Heiden MG (2017). Environmental cystine drives glutamine anaplerosis and sensitizes cancer cells to glutaminase inhibition. eLife.

[33] Wang Y, Yang L, Zhang X, Cui W, Liu Y, Sun QR (2019). Epigenetic regulation of ferroptosis by H2B monoubiquitination and p53. EMBO Rep.

[34] Zhang C, Liu Z, Zhang Y, Ma L, Song E, Song Y (2020). “Iron free” zinc oxide nanoparticles with ion-leaking properties disrupt intracellular ROS and iron homeostasis to induce ferroptosis. Cell Death Dis.

[35] Gu P, Chen X, Xie R, Xie W, Huang L, Dong W (2019). A novel AR translational regulator lncRNA LBCS inhibits castration resistance of prostate cancer. Mol Cancer.

[36] Zhang X, Zhou Y, Chen S, Li W, Chen W, Gu W (2019). LncRNA MACC1-AS1 sponges multiple miRNAs and RNA-binding protein PTBP1. Oncogenesis.

[37] Zhou C, Yi C, Yi Y, Qin W, Yan Y, Dong X (2020). LncRNA PVT1 promotes gemcitabine resistance of pancreatic cancer via activating Wnt/β-catenin and autophagy pathway through modulating the miR-619-5p/Pygo2 and miR-619-5p/ATG14 axes. Mol Cancer.

[38] Liu Z, Mo H, Sun L, Wang L, Chen T, Yao B (2020). Long noncoding RNA PICSAR/miR-588/EIF6 axis regulates tumorigenesis of hepatocellular carcinoma by activating PI3K/AKT/mTOR signaling pathway. Cancer Sci.

[39] Li G, Shi H, Wang X, Wang B, Qu Q, Geng H (2019). Identification of diagnostic long non-coding RNA biomarkers in patients with hepatocellular carcinoma. Mol Med Rep.

[40] Tan T, Li J, Wen Y, Zou Y, Yang J, Pan J (2021). Association between lncRNA-H19 polymorphisms and hepatoblastoma risk in an ethic Chinese population. J Cell Mol Med.

