# Supplementary material for: LncRNA HEPFAL accelerates ferroptosis in hepatocellular carcinoma by regulating SLC7A11 ubiquitination
Source: Cell Death Dis. 2022 Aug 25;13(8):734. doi: 10.1038/s41419-022-05173-1 (PMC9411508; doi:10.1038/s41419-022-05173-1)
Supplement: Supplementary file 1 — Supplementary information [file 41419_2022_5173_MOESM1_ESM.pdf]

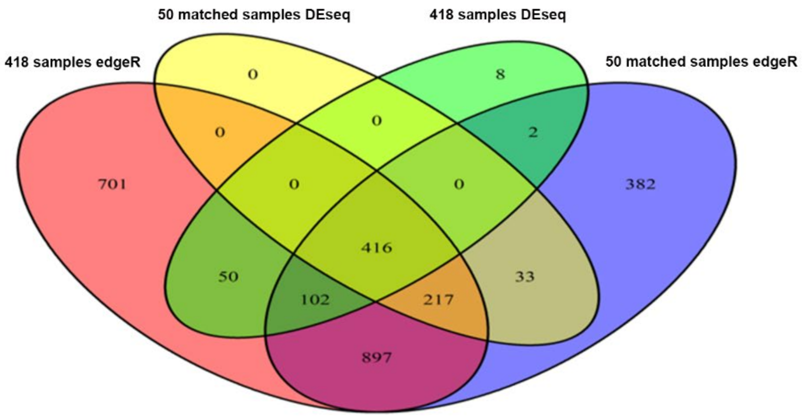

Figure S1. DEseq and edgeR differential expression analysis on 368 samples and 50 paired samples to filter lncRNAs.

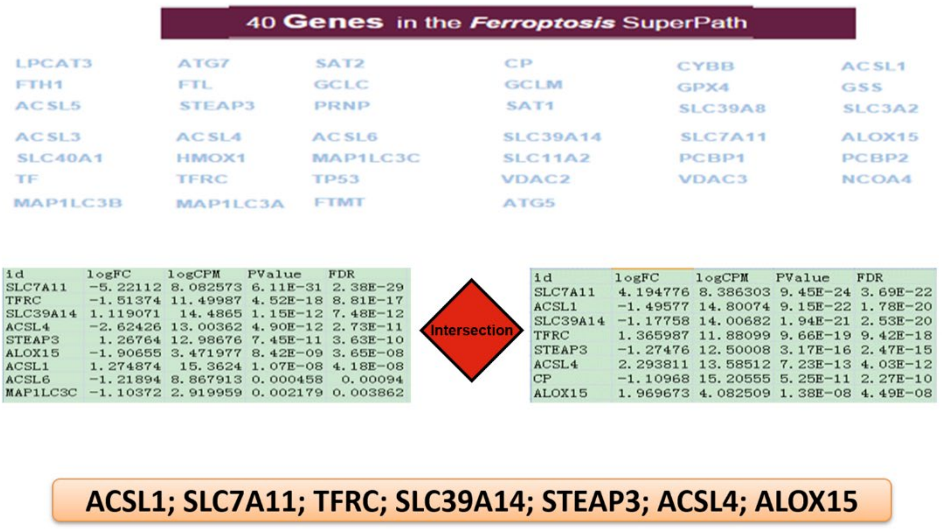

id	logFC	logCPM	PValue	FDR
SLC7A11	-5.22112	8.082573	6.11E-31	2.38E-29
TFRC	-1.51374	11.49987	4.52E-18	8.81E-17
SLC39A14	1.119071	14.4865	1.15E-12	7.48E-12
ACSL4	-2.62426	13.00362	4.90E-12	2.73E-11
STEAP3	1.26764	12.98676	7.45E-11	3.63E-10
ALOX15	-1.90655	3.471977	8.42E-09	3.65E-08
ACSL1	1.274874	15.3624	1.07E-08	4.18E-08
ACSL6	-1.21894	8.867913	0.000458	0.00094
MAP1LC3C	-1.10372	2.919959	0.002179	0.003862

id	logFC	logCPM	PValue	FDR
SLC7A11	4.194776	8.386303	9.45E-24	3.69E-22
ACSL1	-1.49577	14.80074	9.15E-22	1.78E-20
SLC39A14	-1.17758	14.00682	1.94E-21	2.53E-20
TFRC	1.365987	11.88099	9.66E-19	9.42E-18
STEAP3	-1.27476	12.50008	3.17E-16	2.47E-15
ACSL4	2.293811	13.58512	7.23E-13	4.03E-12
CP	-1.10968	15.20555	5.25E-11	2.27E-10
ALOX15	1.969673	4.082509	1.38E-08	4.49E-08

Figure S2. edgeR differential expression analysis on 368 samples and 50 paired samples to filter ferroptosis-related genes.

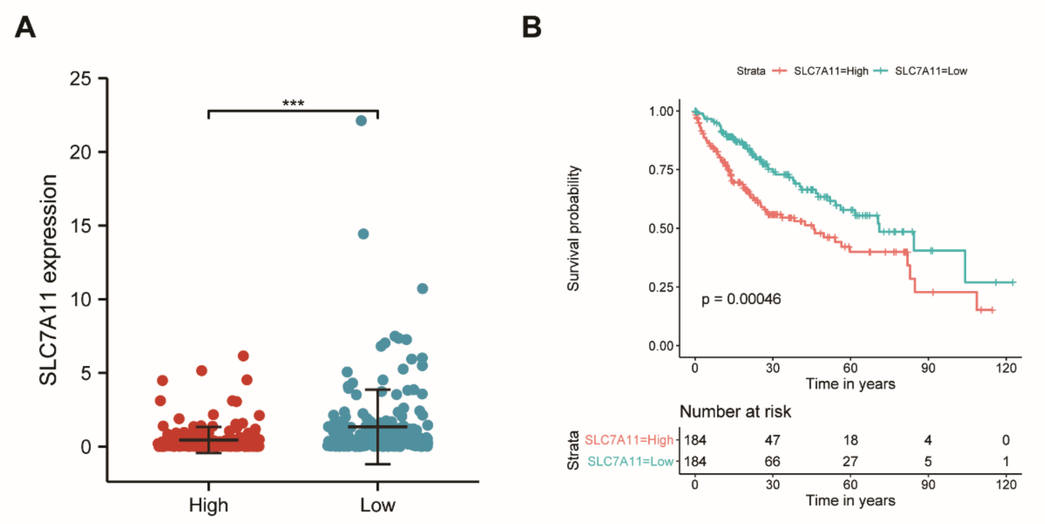

Figure S3. SLC7A11 is associated with LncRNA HEPFAL and affects the prognosis of patients. **A)** Expression of SLC7A11 in high and low expression LncRNA tissues. **B)** SLC7A11 affects OS in HCC patients.

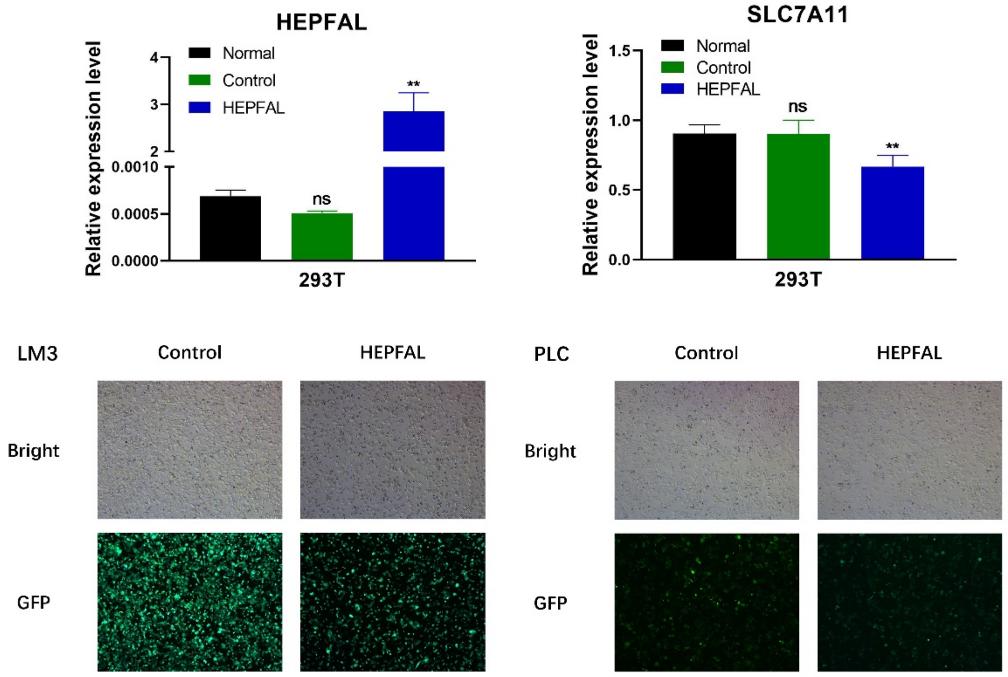

Figure S4. Relative expression of lncRNA HEPFAL and SLC7A11 in 293T cells transfected with lncRNA HEPFAL expression vector and control vector was detected by qRT-PCR. And GFP fluorescence in cells after overexpression lncRNA HEPFAL.

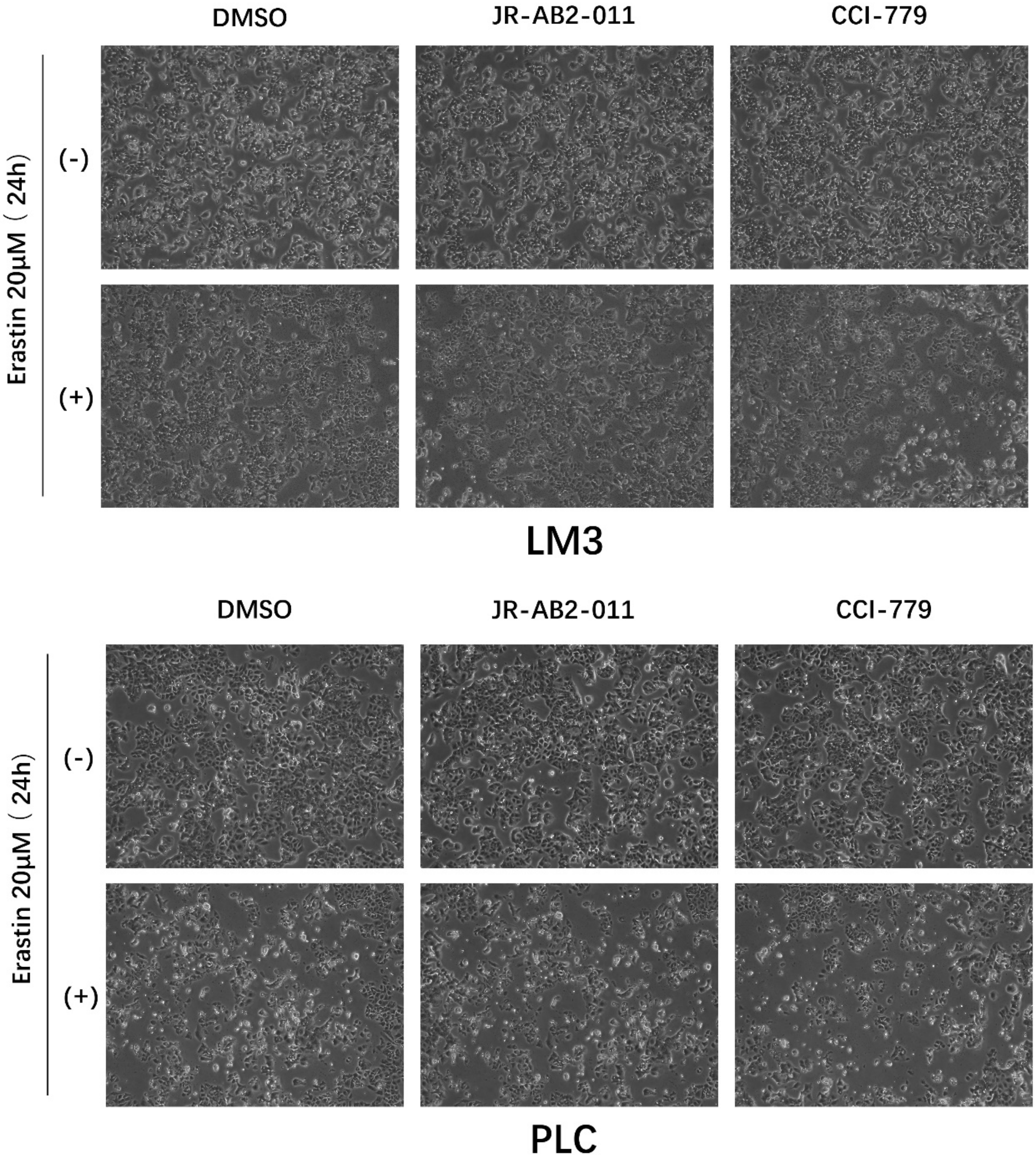

Figure S5. HCCLM3 and PLC cells were treated with 20μM erastin or untreated and treated with DMSO, JR-AB2-001 and CCI-779, respectively.

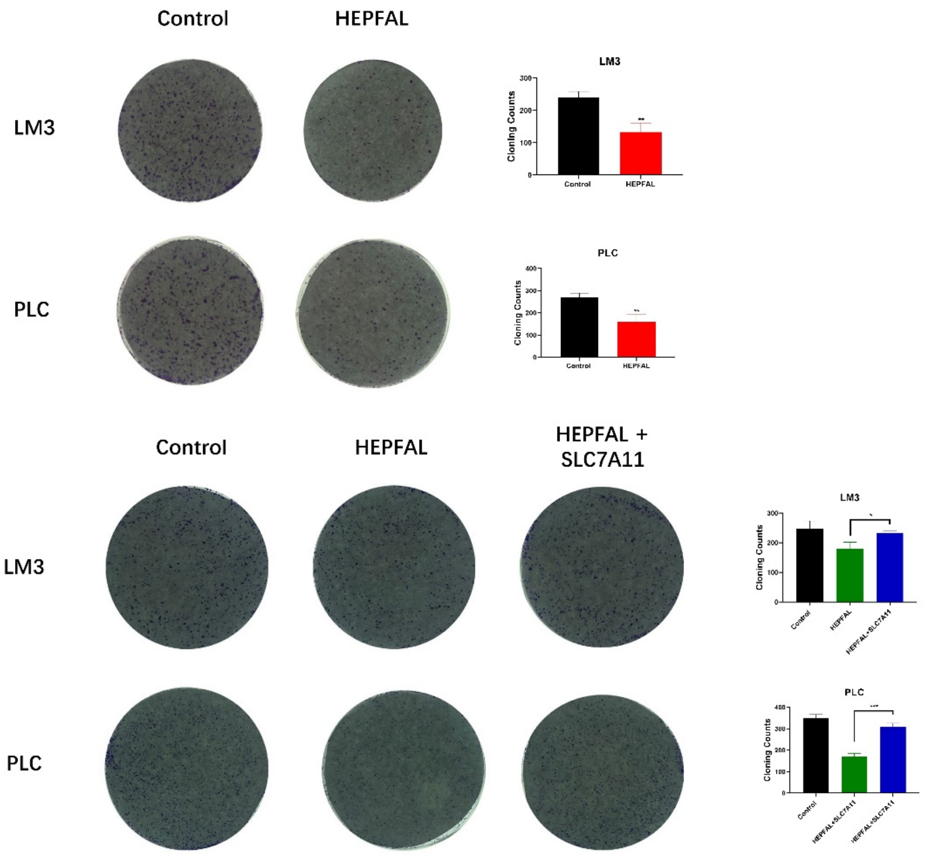

Figure S6. the number of colonies of LM3 and PLC cells after transfected as indicated.

Your input sequence is:

TAGACCACAGGGAGGCCTGGGAACTGCCTGAGCACCTGCACCA
CGGGAGA GGCTTGGGTGACACTTGGGCACCTGCACCCAACACA
GGTAGCAGTGATCA CAGTGAAAACATTTTGTTAGCAGTCGTCTGC
TTCCTGTCCTGGCATGAGC AGCAACGGGTAAAGGCACTCTTGT
CAGCTGAGGGGATGAGTGGGGCTCT GCCCAGCTGGTTGGAGAC
TCCAGCAGGTTTGCAGGAGGAGACGGAAGAGC AAGGAGTGGAG
GCAAAAACTGCTGCCAAGATCCTGTGGGAAAACAGGAAC CCAAA
GAAGGAACTCCAATGGGAAACTAATTTAGACAACAATGATCTTAA A
GTTTTTGCTCCTTCGAGACTGTTGAAAAGAACTCCCACGAGTGAA
CAAA AGACGCCTGGCCGAGGACACTCCAGAGTGCTGTCACGAG
GGTTCCCTGCA GTTTTCAGAAACTTGGAGCATGTGGCCACAGCG
GCTTGCACGTGATTCAT ACGGCACATCTTGTCACCATGTAAATGTT
TATCAGCAGAGACAAAGACCA GAAGAGCAACCTTGGAAAGAGCT
AAATATTCCGATGTTCCAGCATTTTCC CCTCTGGTTTGTATTGTGA
ATTTTTAAAATCTCGATTGACAAGT

------------ lncLocator Prediction Result ------------

Subcellular locations	score
Cytoplasm	0.585413276878
Nucleus	0.0907816604298
Ribosome	0.101696991218
Cytosol	0.130737666977
Exosome	0.0913704044975

Predicted location
Cytoplasm

Figure S7. The predicted location of lncRNA HEPFAL.

Table S1. Sequences of the primers used for quantitative real-time PCR.

Gene	Forward primer(5’-3’)	Reverse primer(5’-3’)
SLC7A11	CCATGAACGGTGGTGTGTT	GACCCTCTCGAGACGCAAC
lncRNA HEPFAL	CGGGTAAAGGGCACTCTTGTCAG	TCCACTCCTTGCTCTTCCGTCTC
GAPDH	CAAGGTCATCCATGACAACTTTG	GTCCACCACCCTGTTGCTGTAG
