# Supplementary material for: LncRNA HEPFAL accelerates ferroptosis in hepatocellular carcinoma by regulating SLC7A11 ubiquitination
Source: Cell Death Dis. 2022 Aug 25;13(8):734. doi: 10.1038/s41419-022-05173-1 (PMC9411508; doi:10.1038/s41419-022-05173-1)
Supplement: Supplementary file 2 — original western blots [file 41419_2022_5173_MOESM2_ESM.pdf]

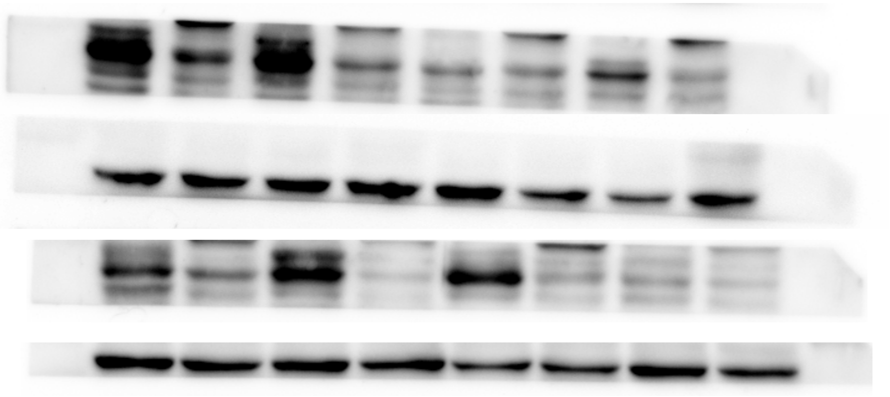

Figure S8. original western blots of Figure 1I.

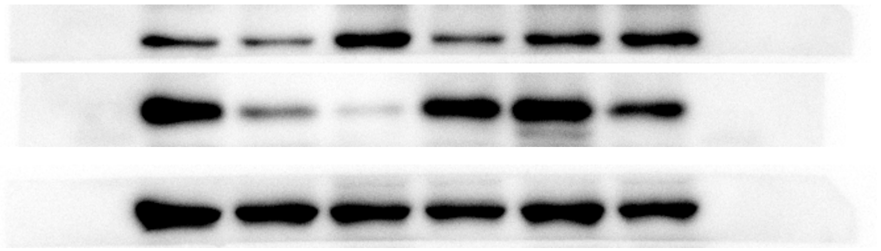

Figure S9. original western blots of Figure 2C.

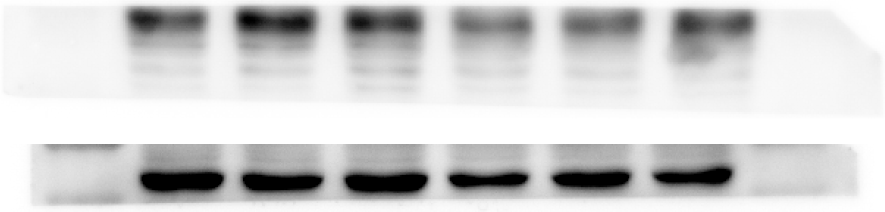

Figure S10. original western blots of Figure 2F.

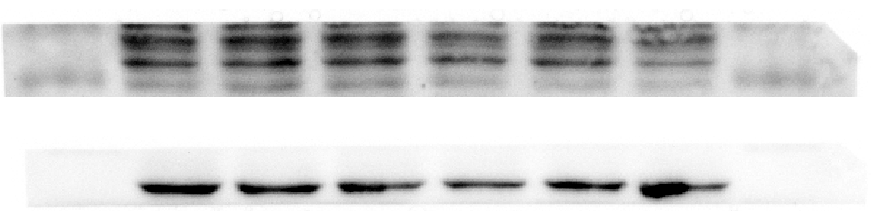

Figure S11. original western blots of Figure 2I.

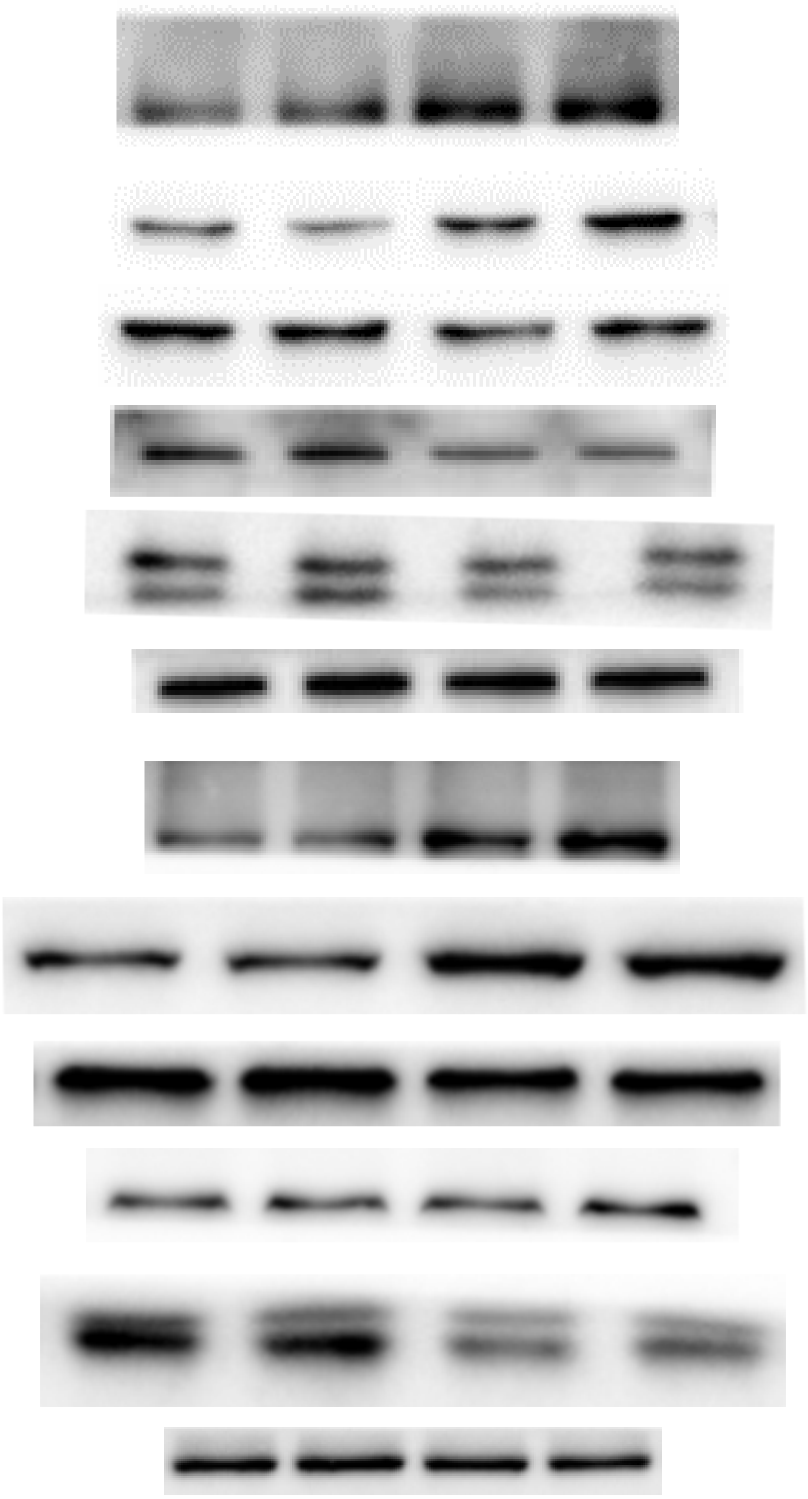

Figure S12. original western blots of Figure 3E.

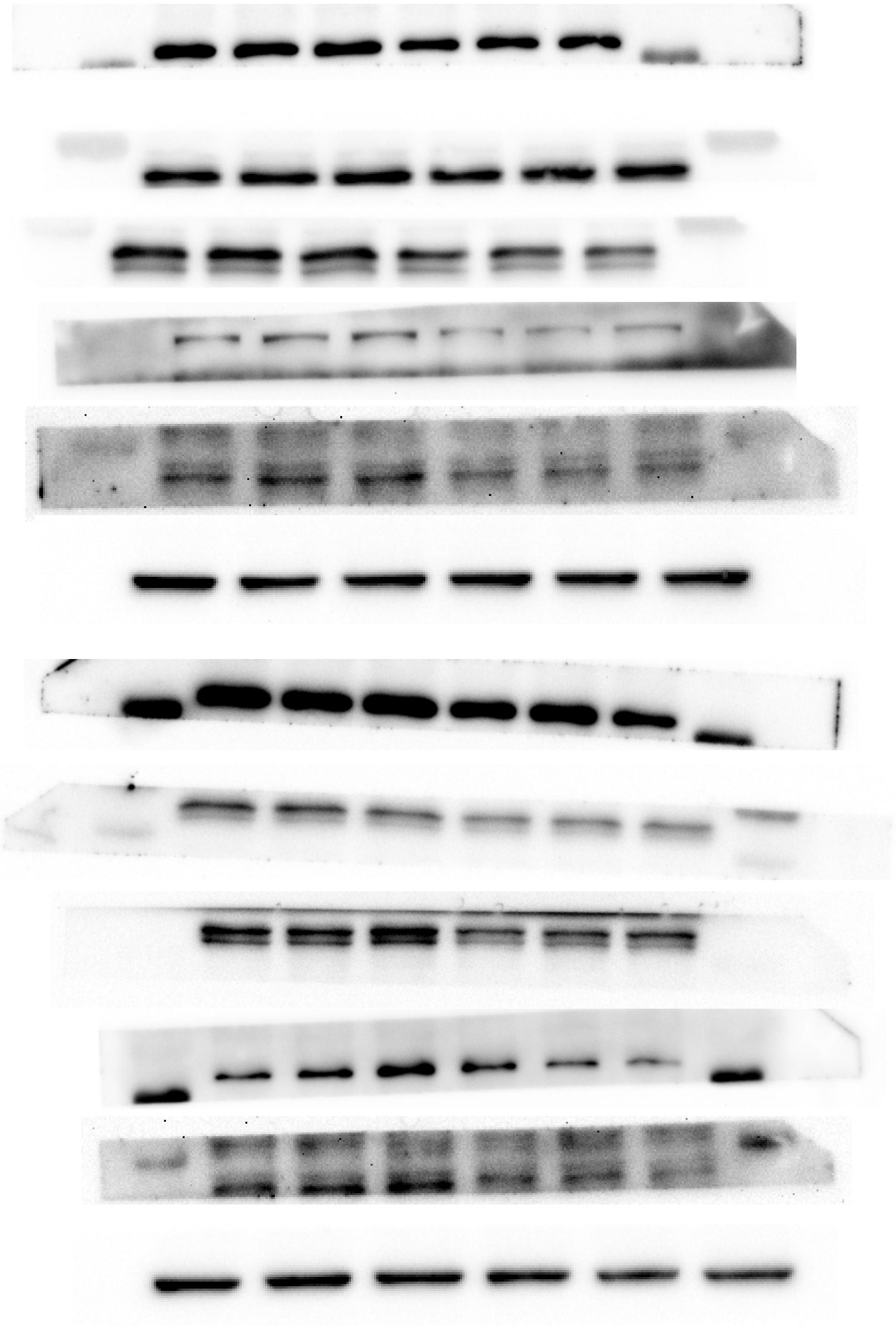

Figure S13. original western blots of Figure 4H.

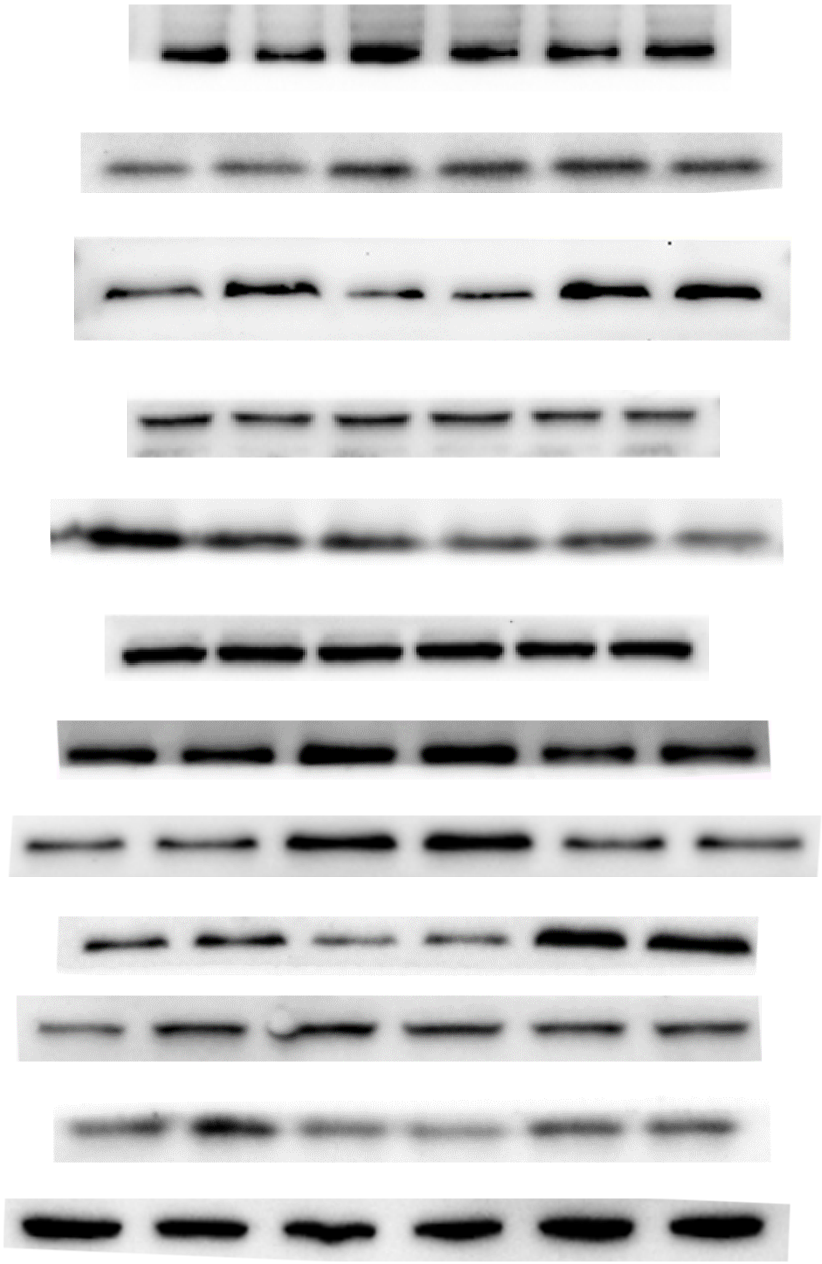

Figure S14. original western blots of Figure 5H.

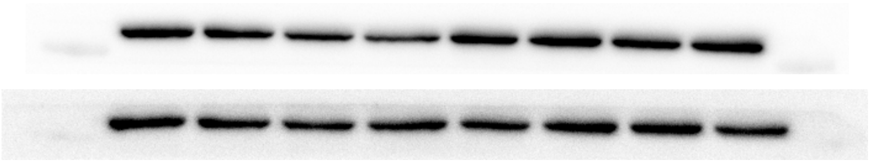

Figure S15. original western blots of Figure 6B.

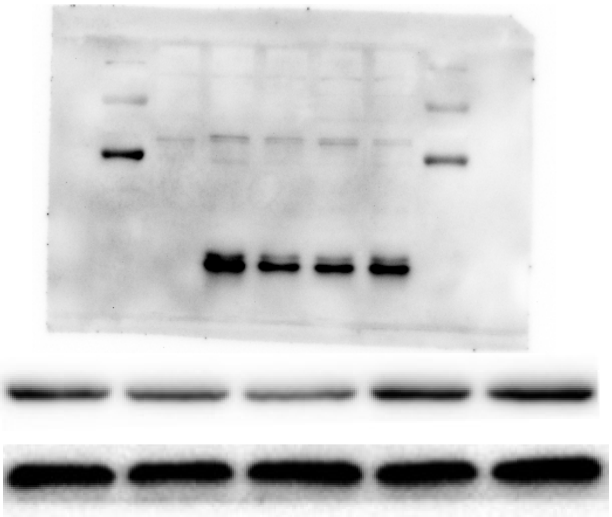

Figure S16. original western blots of Figure 6C.

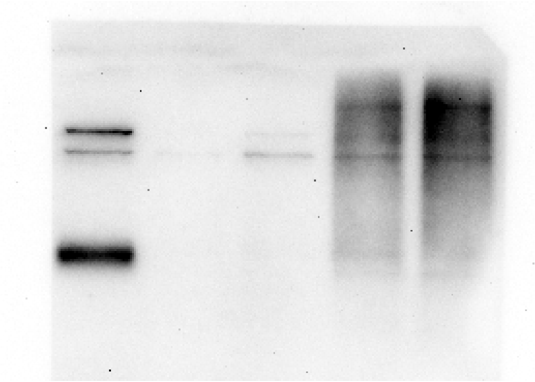

Figure S17. original western blots of Figure 6D.

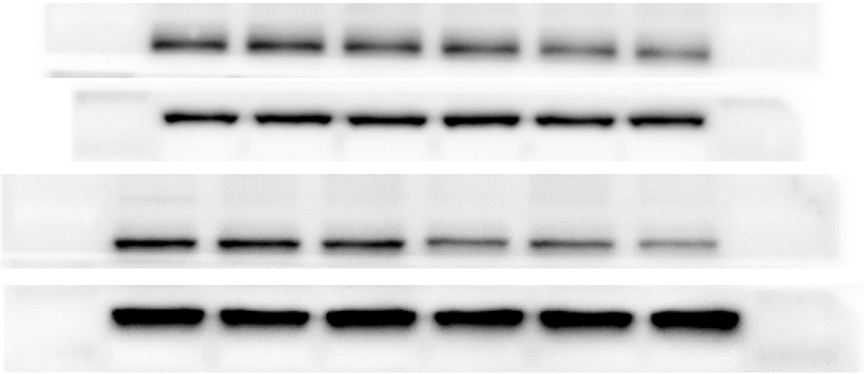

Figure S18. original western blots of Figure 6E.

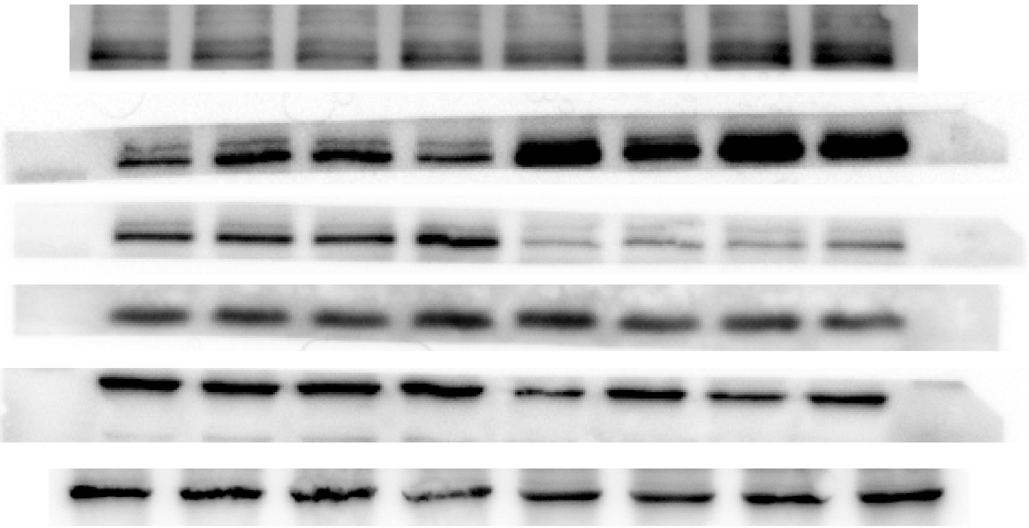

Figure S19. original western blots of Figure 7H.

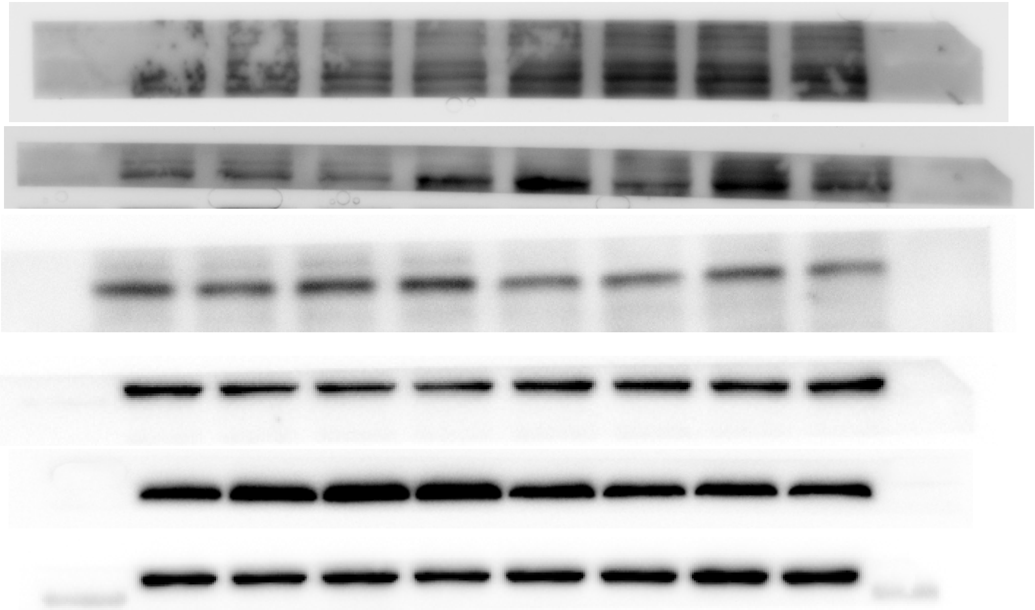

Figure S20. original western blots of Figure 7I.
